# Change in Oxidative Stress and Mitochondrial Dynamics in Response to Elevated Cold-Inducible RNA-Binding Protein in Cardiac Surgery-Associated Acute Kidney Injury

**DOI:** 10.1155/2022/3576892

**Published:** 2022-07-09

**Authors:** Xinglong Zheng, Yang Fan, Jing Li, Tao Ma, Yongxin Li, Qian Wang, Yang Yan, Wenyan Liu

**Affiliations:** ^1^Department of Cardiovascular Surgery, First Affiliated Hospital of Xi'an Jiaotong University, Xi'an, Shaanxi, China; ^2^Xi'an Medical University, Xi'an, Shaanxi, China; ^3^Department of Operation and Anesthesia, First Affiliated Hospital of Xi'an Jiaotong University, Xi'an, Shaanxi, China; ^4^Department of Blood Purification, First Affiliated Hospital of Xi'an Jiaotong University, Xi'an, Shaanxi, China

## Abstract

Cardiac surgery-associated acute kidney injury (CSA-AKI) is a common yet serious complication that is closely related to cardiopulmonary bypass (CPB). Extracellular cold-inducible RNA-binding protein (eCIRP) can mediate aseptic inflammation and trigger intracellular oxidative stress. In the present study, expression of serum CIRP was significantly elevated post-CPB (785.0 ± 640.5 pg/mL vs. 149.5 ± 289.1 pg/mL, *P* < 0.001) and was positively correlated with CPB duration (*r* = 0.502, *P* < 0.001). Patients with high expression of CIRP had higher risks of postoperative AKI than patients with low CIRP expression (OR: 1.67, 95% CI 1.04-2.68). In a rat CPB model, the serum CIRP concentration increased significantly after CPB. Similarly, the levels of Scr and BUN significantly increased 4 hours after CPB. KIM-1 and NGAL mRNA levels in the CPB group were 8.2 and 4.3 times higher than the sham group, respectively. In addition, the levels of inflammatory cell infiltration, oxidative stress, and apoptosis in the renal tissue of the CPB group were significantly higher compared to the sham group. The expression levels of serum inflammatory factors at 4 hours post-CPB were also increased. Administration of recombinant human CIRP protein promoted the expression of NADPH oxidase via the TLR-4/MyD88 pathway, aggravated intracellular oxidative stress, mediated mitochondrial dynamics disorder, and eventually increased apoptosis in HK-2 cells. However, the CIRP inhibitor C23 improved the CIRP-mediated oxidative stress and mitochondrial dysfunction in both rat and cell models. In summary, elevated CIRP could mediate oxidative stress and mitochondrial dynamics in the kidney to promote CSA-AKI.

## 1. Introduction

As the second most common cause of acute kidney injury (AKI) in the intensive care unit (ICU), cardiac surgery-associated AKI (CSA-AKI) is linked to elevated short- and long-term mortality and morbidity [1–3]. CSA-AKI's pathophysiology is multifactorial and likely includes perioperative renal ischemia-reperfusion injury (IRI), hemolysis and pigment nephropathy induced by cardiopulmonary bypass (CPB), oxidative stress, and inflammation [4]. Commonly known as an independent risk factor for CSA-AKI [5], CPB not only can cause oxidative stress [6, 7] but can also change hemodynamic status [8].

Low levels of cold-inducible RNA-binding protein (CIRP) in various tissues can be induced by stress (e.g., hypothermia and hypoxia) [9]. Extracellular CIRP (eCIRP), functioning as a damage-associated molecular pattern (DAMP), can trigger proinflammatory responses [10]. Studies have demonstrated that blocking CIRP secretion can effectively reduce IRI in the liver and kidney [11, 12]. During cardiac surgery, a patient's body temperature usually drops to approximately 30°C, and the temperature during an aortic dissection surgery is even lower. CPB provides nonpulsatile blood flow and causes a relatively low perfusion of all organs. During this process, high levels of CIRP may be released into the circulation.

Intracellular reactive oxygen species (ROS) are mainly caused by nicotinamide adenine dinucleotide phosphate (NADPH) oxidase-produced superoxide anion [13]. Li et al. found that CIRP released from damaged tissue could induce NADPH oxidase-derived ROS via the TLR-4/MyD88 signaling pathway to promote fragmentation of mitochondrial DNA [14]. Mitochondrial fusion and fission are two opposing processes that together regulate mitochondrial dynamics [15, 16]. ROS accumulation in cells can lead to mitochondrial dynamics disorders and promote mitochondrial fission by upregulating dynamin-related protein 1 (Drp1) and fission 1 (Fis1) expression. Overwhelmed mitochondrial fission subsequently leads to the fragmentation of mitochondrial DNA and repeated ROS generations [17]. This vicious cycle between oxidative stress and disturbed mitochondrial dynamics may further intensify tissue damage.

Our previous study demonstrated that CIRP serum levels were significantly elevated after CPB, which was closely related to the occurrence of AKI [18]. Therefore, we hypothesized that excessive CIRP is secreted into the circulation during CPB, which may subsequently induce oxidative stress and disturb mitochondrial dynamics to promote CSA-AKI.

## 2. Methods

### 2.1. Patient Enrollment and Clinical Specimens

The Ethics Committee of the First Affiliated Hospital of Xi'an Jiaotong University approved this study. Prior to the study, all patients gave written informed consent. Different types of cardiac surgeries included coronary artery bypass grafting (CABG), valve replacement, and aortic dissection for congenital heart disease. At the end of the anesthesia and surgery, serum specimens were obtained from each patient and stored at -80°C until further analysis. The criteria of the Kidney Disease Improving Global Outcomes Definition and Staging (KDIGO) was used to define AKI that occurred within 7 days postsurgery. The CPB time duration for each patient was recorded.

### 2.2. Rat CPB Model

The CPB circuit consisted of a peristaltic pump (LONGER, China), a hollow fiber oxygenator (Xijian Medical, China), a heat exchanger (Xijian Medical), and an open bath thermostat (HerryTech, China). Before conducting the experiment, the system was primed with about 8 mL of hydroxyethyl starch. This study used male Sprague-Dawley rats aged 14-16 weeks and weighing 400-450 g. The Animals Care and Use Committee of Xi'an Jiaotong University approved all animal protocols. The rats were randomly divided into three groups: sham (*n* = 5), CPB (*n* = 6), and CPB+C23 (C23) (*n* = 7). The sham group was only cannulated and slowly injected with 8 mL of hydroxyethyl starch, while the C23 group was intraperitoneally injected with the CIRP inhibitor C23 (8 mg/kg, Bioyears, China) 30 minutes before surgery.

CPB was performed as previously reported [19, 20]. The rats were anesthetized with isoflurane and intubated. The 20-Gauge (20 G) and 14 G catheters were then cannulated in the tail artery and right external jugular vein, respectively. At the same time, a 24 G catheter was cannulated in the branch of the left femoral artery to monitor the arterial blood pressure. A thermometer was placed into the rectum of the rats. After the injection of 1 mL of heparin saline (250 IU/mL), CPB was started with a flow rate of 120-140 mL/kg/min. The average pressure was maintained at 70-90 mmHg, and the temperature was maintained between 26 and 28°C. The duration of the entire CPB was 60 minutes. We saved 1 mL of arterial blood, removed the intubation, and sutured the incision. The rats were sacrificed 4 hours after the surgery, and the serum and renal tissues were obtained.

### 2.3. Cell Culture

Human Kidney 2 (HK-2) cells were cultured in DMEM (Gibco) supplemented with 10% fetal bovine serum (FBS) (Gibco) and 1% pen/strep (HyClone). The cells were cultured in 5% CO_2_ at 37°C and treated with recombinant human CIRP protein (rhCIRP, Cloud-Clone Crop, China) or C23 for 6 hours. The cells were divided into four groups: control (*n* = 3), low rhCIRP (100 ng/mL) (*n* = 3), high rhCIRP (1000 ng/mL) (*n* = 3), and high rhCIRP (1000 ng/mL)+C23 (300 ng/mL) (*n* = 3).

### 2.4. Enzyme-Linked Immunosorbent Assays (ELISA)

The CIRP concentrations in the human and rat serum were detected using CIRP ELISA kits (SEG886Hu and SEG886Ra, Cloud-Clone Corp.) according to the manufacturer's protocol. The concentration difference of CIRP in the human serum between the two time points was recorded as *Δ*CIRP. The ELISA kits for interleukin 6 (IL-6, E-EL-H0102c and E-EL-R0015c, Elabscience), IL-1*β* (E-EL-H0149c and E-EL-R0012c, Elabscience), and tumor necrosis factor alpha (TNF-*α*, E-EL-H0109c and E-EL-R2856c, Elabscience) were used to detect inflammatory factors in the rat serum and the cell culture medium.

### 2.5. Histological Biochemical Analyses

Renal tissue samples were embedded in paraffin after fixation with 4% paraformaldehyde and then cut into 5 *μ*m sections for hematoxylin and eosin (H&E) staining. A urea assay kit (C013-2, Nanjing Jiancheng Bioengineering Institute) and creatinine (Cr) assay kit (sarcosine oxidase) (C011-2, Nanjing Jiancheng Bioengineering Institute) were used to detect blood urea nitrogen (BUN) and serum creatinine (Scr).

### 2.6. TUNEL Staining and Apoptosis Assays

Cell apoptosis in the renal tissues of each group was measured using the DeadEnd Fluorometric TUNEL System (Promega) according to the manufacturer's protocol. Through a fluorescence microscope, the sections were analyzed by choosing representative fields for application. The apoptotic HK-2 cells treated with rhCIRP or C23 were assayed using the apoptosis detection kit (A211-02, Vazyme) and then analyzed by flow cytometry (cytoFLEX, Beckman Coulter). The percentage of the apoptotic cells was calculated using early and late apoptotic cells.

### 2.7. Quantitative Real-Time Reverse Transcription Polymerase Chain Reaction (RT-PCR)

Kidney injury molecule 1 (KIM-1) and urinary neutrophil gelatinase-associated lipocalin (NGAL) were used to evaluate renal injury. Total RNA of the renal tissues and the HK-2 cells was isolated using TRIzol (RR037A, Takara). mRNA levels of KIM-1 and NGAL were normalized to *β*-actin. The following primer sets were used: rat KIM-1: forward 5′-ACCAAGTCACCTATCGGAGC-3′ and reverse 5′-TGTTGGAGG ACTTGTGGGAA-3′, for rat NGAL: forward 5′-CGAATGCGGTCCAGAAAGAA-3′ and reverse 5′-CCACTTGCACATCGTAGCTC-3′, for Homo sapiens KIM-1: forward 5′-CACCCAAAAGAGCAAGAAGCA-3′ and reverse 5′-CCTCAGCC AGCAGAAACCC-3′, and for Homo sapiens NGAL: forward 5′-GCTGGTTGTAGTTGGTGC-3′ and Homo sapiens NGAL: reverse 5′-CAGGGGAAGTGGTATGTG-3′. The comparative-Ct method (*ΔΔ*Ct method) was used to calculate the relative mRNA levels.

### 2.8. Immunohistochemistry

Immunohistochemical staining was performed on the paraffin-embedded renal tissue sections. Infiltration of neutrophils and macrophages was shown by myeloperoxidase (MPO, 22225-1-AP, Proteintech) and F4/80 (28463-1-AP, Proteintech) staining, respectively.

### 2.9. Oxidative Stress Measurement

Malonaldehyde (MDA), glutathione peroxidase activity (GSH-Px), and superoxide dismutase (SOD) from the renal tissue homogenate were measured using assay kits (A003-1, A005, and A001-1, Nanjing Jiancheng Bioengineering Institute). Dihydroethidium (DHE) dye (D7008, Sigma-Aldrich) was used to detect ROS in the HK-2 cells. The ImageJ software was used to quantify the fluorescence intensity.

### 2.10. Western Blot Assay

Using 7.5-15% sodium dodecylsulfate-polyacrylamide gel electrophoresis (SDS-PAGE), proteins were fractionated and then transferred onto polyvinylidene fluoride (PVDF) membranes (Millipore, Germany). The PVDF membranes were incubated with the primary antibodies at 4°C overnight, followed by incubation with horseradish peroxidase- (HRP-) conjugated secondary antibodies. Apoptosis was indexed by measuring expression of anti-Bax (MA5-32031, Invitrogen, 1 : 1000), anti-Bcl2 (DF6015, Affinity, 1 : 500), and anti-caspase-3 (ab13847, Abcam, 1 : 500). NADPH oxidase was detected using anti-gp91^phox^ (19013-1-AP, Proteintech, 1 : 500) and anti-p47^phox^ (YT3520, Immunoway, 1 : 500). Mitochondrial dynamics was determined by measuring expression of anti-Fis1 (10956-1-AP, Proteintech, 1 : 1000), anti-Drp1 (DF7037, Affinity, 1 : 500), and anti-Mfn2 (12186-1-AP, Proteintech, 1 : 2000). The other antibodies used in this study included TLR-4 (19811-1-AP, Proteintech, 1 : 1000), MyD88 (BA2321, Boster, 1 : 500), and anti-*β*-actin (4967, Cell Signaling Technology, 1 : 1000). Clarity Western ECL substrate (Bio-Rad Laboratories) and a Universal Hood III imaging system (Bio-Rad) were used to detect the proteins, respectively.

### 2.11. Statistical Analysis

All measurement data are presented as means ± standard deviation (SD). The correlation between the CPB time and the *Δ*CIRP was determined using linear regression analysis with the Pearson's test. Differences among the groups were analyzed by *T*-test or one-way ANOVA using SPSS 25 (IBM Corp., NY). Statistical significance was accepted at *P* < 0.05.

## 3. Results

### 3.1. Serum CIRP Expression and AKI Incidence after CPB

**Figure 1:**
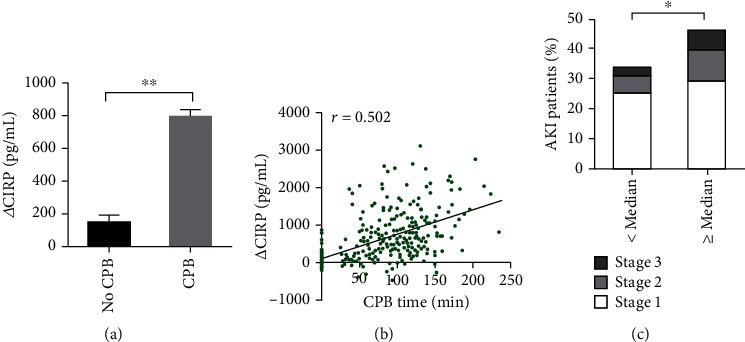
Elevated CIRP after CPB was associated with increased incidence of AKI. (a) The *Δ*CIRP levels in patients who experienced CPB were 4.3-fold higher than those who did not. (b) The *Δ*CIRP levels were positively correlated with the CPB time (*n* = 292, *r* = 0.502, and *P* < 0.001). (c) Incidence of AKI stratified by median serum *Δ*CIRP concentration. CIRP: cold-inducible RNA-binding protein; CPB: cardiopulmonary bypass. ^∗^*P* < 0.05; ^∗∗^*P* < 0.001.

A total of 292 paired serum specimens were collected from patients who underwent cardiac surgeries between May 1, 2020 and September 30, 2020, of which 249 patients underwent CPB and 43 patients did not (Baseline characteristics of the study patients were listed in Table 1 in supplementary files). The *Δ*CIRP values of the patients who underwent CPB were significantly higher than those without (785.0 ± 640.5 pg/mL vs. 149.5 ± 289.1 pg/mL, *P* < 0.001, Figure 1(a)). Correlation analysis revealed that the *Δ*CIRP values and the CPB time were positively correlated (*r* = 0.502, *P* < 0.001, Figure 1(b)). Compared to patients with low serum *Δ*CIRP (below the median value), patients with high *Δ*CIRP showed an increased incidence of postoperative AKI (34% vs. 47%, odds ratio (OR):1.67, 95% confidence interval (CI) 1.04-2.68, and *P* = 0.032). Specifically, patients with high *Δ*CIRP had a significantly higher incidence of severity (stage 2 or stage 3) for AKI (17%) compared to patients with low *Δ*CIRP (9%) (*P* = 0.037, Figure 1(c)).

### 3.2. CIRP Secretion and Renal Injury after CPB

**Figure 2:**
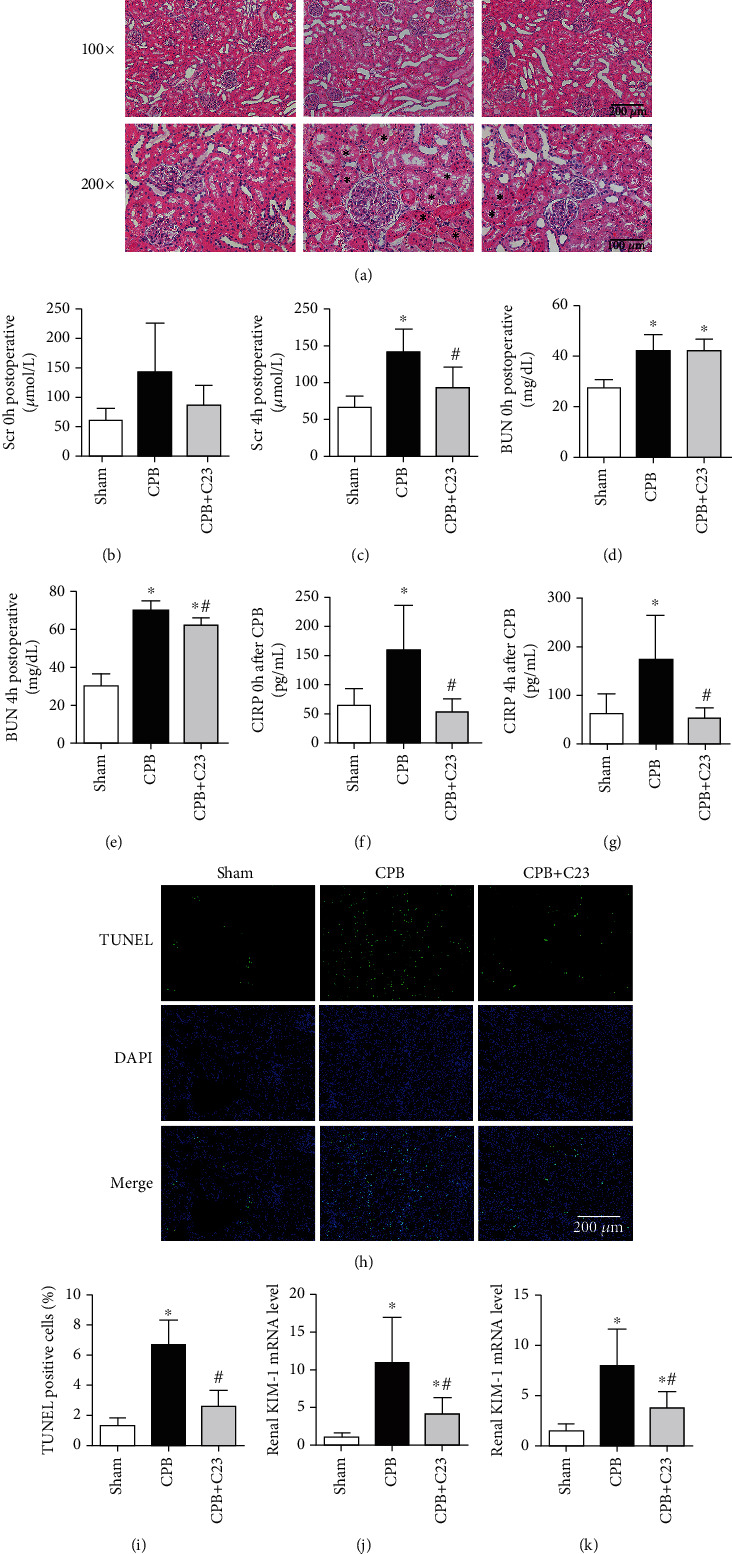
CPB promoted CIRP secretion and aggravated renal injury. (a) H&E staining showing more dilated renal tubules and tube casts in the CPB group compared to the sham group and CPB+C23 group. (b, c) Expression levels of Scr at 0 and 4 hours after CPB. (d, e) Expression levels of BUN at 0 and 4 hours after CPB. (f, g) Expression levels of CIRP at 0 and 4 hours after CPB. (h, i) TUNEL staining indicating the increased number of apoptotic cells in the renal tissue after CPB; C23 administration effectively reduced apoptosis (magnification 200x). (j, k) The mRNA levels of renal injury markers KIM-1 and NGAL in renal tissues. CIRP: cold-inducible RNA-binding protein; CPB: cardiopulmonary bypass; Scr: serum creatinine; BUN: blood urea nitrogen. ^∗^*P* < 0.05 vs. the sham group; ^#^*P* < 0.05 vs. CPB. (a, h, top) Scale bar: 200 *μ*m and (a, bottom) scale bar: 100 *μ*m.

**Figure 3:**
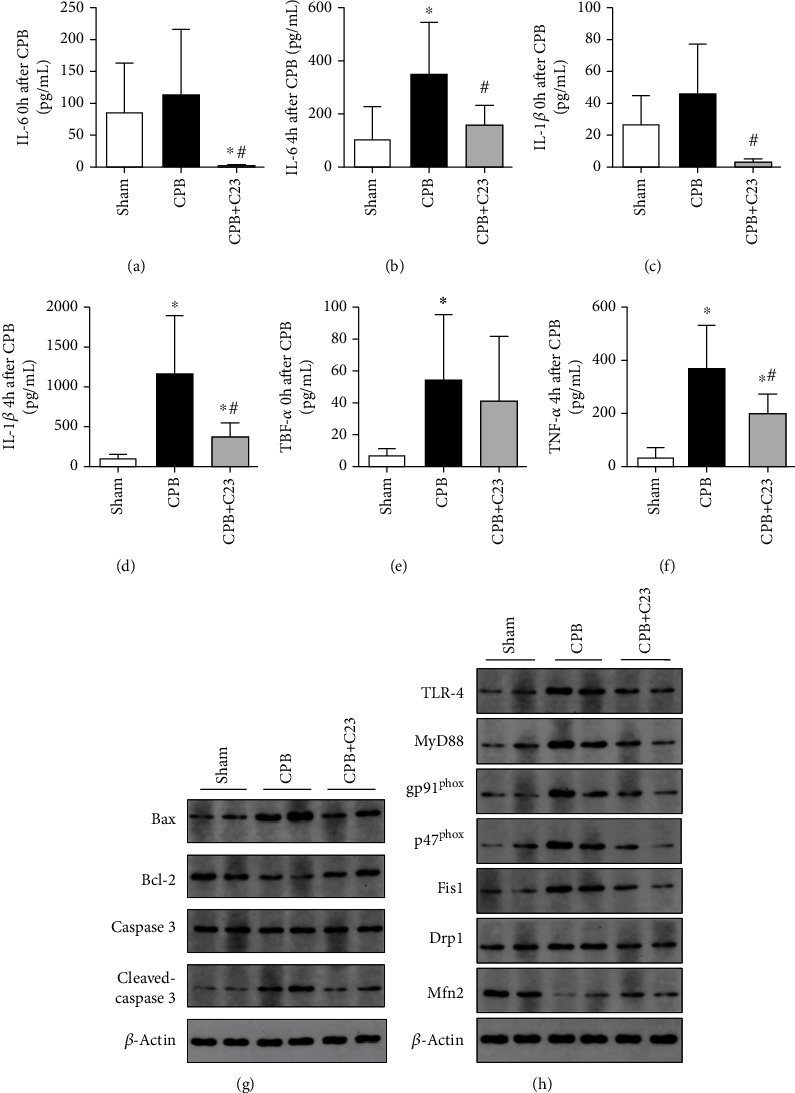
CPB induced mitochondrial dynamics disorder and increased inflammatory factor secretion. (a–f) Expression of IL-6, IL-1*β*, and TNF-*α* in the serum at 0 and 4 hours after CPB. Compared to the CPB group, C23 administration decreased the expression of IL-6, IL-1*β*, and TNF-*α* by 54.0%, 67.5%, and 45.2% at 4 hours, respectively. (g) Western blot analysis of renal Bax, Bcl-2, and cleaved-caspase-3 expression. (h) CPB upregulated the NADPH oxidase via the TLR-4/MyD88 pathway, which promoted the expressions of mitochondrial fission-related proteins Fis1 and Drp1 and reduced the expression of fusion-related protein Mfn2. ^∗^*P* < 0.05 vs. sham; ^#^*P* < 0.05 vs. CPB.

A rat CPB model was established to assess the effect of CPB on the kidney. H&E staining was used to evaluate renal histological injury. Compared to the sham group, renal tubules in the CPB group were dilated and tube-casted. Scr and BUN showed significant increases at 4 hours after CPB, but they decreased when treated with C23 (*P* < 0.05, Figures 2(b)–2(e)). The concentration of serum CIRP increased significantly after CPB by 2.5-fold of the sham group and slowly increased to 2.8-fold at 4 hours after CPB (*P* < 0.05, Figures 2(f) and 2(g)). However, C23 effectively inhibited the secretion of CIRP. TUNEL staining was used to detect renal cell apoptosis and the counts of TUNEL-positive cells in the CPB group, which were 4.8 times that of the sham group (*P* < 0.05, Figures 2(h) and 2(i)). Moreover, apoptosis genes cleaved caspase-3 and Bax protein expression were higher in the CPB group than the sham group, while expression of apoptosis suppressive gene Bcl-2 was low in the CPB group (Figure 3(g)). KIM-1 and NGAL are novel biomarkers for AKI prediction in clinical practice. mRNA levels of KIM-1 and NGAL in the CPB group were 8.2 times and 4.3 times higher than the sham group, respectively (*P* < 0.05, Figures 2(j) and 2(k)) (raw data of Figure 2 was listed in supplementary files). However, CIRP administration effectively reduced apoptosis in the renal tissue and decrease the risk of AKI.

### 3.3. Renal Oxidative Stress and Mitochondrial Dynamics Disorders after CPB

**Figure 4:**
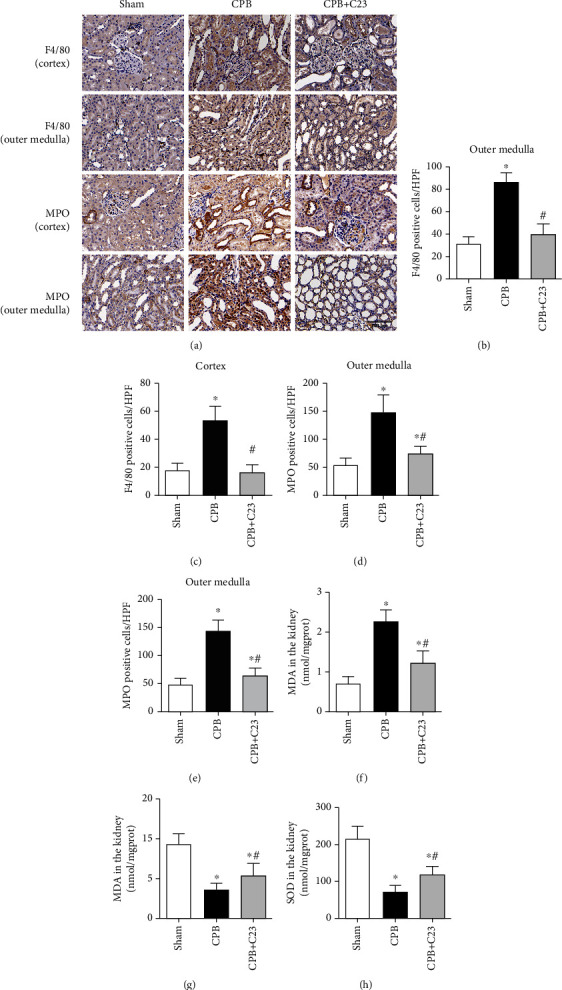
CPB aggravated inflammatory cell infiltration and renal oxidative stress. (a) Immune cell infiltration of macrophages and neutrophils in rat renal tissue at 4 hours after CPB (magnification 400x); quantification of inflammatory cells in renal tissue after CPB; CPB aggravated immune cell infiltration in both the outer medulla and cortex. (b) F4/80-positive macrophages in the outer medulla. (c) F4/80-positive macrophages in the cortex. (d) MPO-positive neutrophils in the outer medulla. (e) MPO-positive neutrophils in the cortex. (f–h) MDA, GSH, and SOD levels in the renal tissue of each group. CPB: cardiopulmonary bypass; MDA: malonaldehyde; GSH: glutathione; SOD: superoxide dismutase. ^∗^*P* < 0.05 vs. sham; ^#^*P* < 0.05 vs. CPB. (a) Scale bars: 200 *μ*m.

In line with the more severe renal injury, tissues in the CPB group showed significantly aggravated inflammatory cell infiltration at 4 hours after CPB. The MPO-positive neutrophils in the CPB group were 3 times that of the sham group in the cortex (*P* < 0.05, Figure 4(d)) and 2.8 times that in the outer medulla (*P* < 0.05, Figure 4(e)). Similarly, the F4/80 positive macrophage in the CPB group was 2.8 times that of the sham group in the cortex (*P* < 0.05, Figure 4(c)) and 2.8 times that in the outer medulla (*P* < 0.05, Figure 4(d)). At the same time, oxidative stress in the renal tissues increased significantly after CPB. Compared to the sham group, CPB increased the renal MDA level but decreased the levels of its reverse indicators, glutathione peroxidase (GSH-Px) and SOD (*P* < 0.05, Figures 4(f)–4(h)). Western blot analysis also indicated that the expression of NADPH oxidase (gp91^phox^ and P47^phox^) was upregulated in the CPB group (Figure 3(h)). CIRP treatment alleviated the inflammatory cell infiltration and reduced the level of oxidative stress (*P* < 0.05).

No difference was observed between the sham group and the CPB group on IL-6 and IL-1*β* expressions at 0 hour after CPB (*P* = 0.623 and *P* = 0.241). IL-6, IL-1*β*, and TNF-*α* expression levels in the serum at 4 hours after CPB were 2.1 times, 24.3 times, and 5.8 times higher than the CPB group at 0 hour after CPB, respectively, and significantly higher compared to the sham group at 4 hours after CPB (*P* < 0.05, Figures 3(a)–3(f)). However, C23 administration reduced the expressions of IL-6, IL-1*β*, and TNF-*α* by 54.0%, 67.5%, and 45.2%, respectively. In addition, CPB promoted the expression of proteins in the TLR-4/MyD88 pathway. Furthermore, the mitochondrial fission-related proteins, Fis1 and Drp1, were expressed at significantly higher levels in the CPB group compared to the sham group. Expression of fusion-related protein, Mfn2, was low in the CPB group (Figure 3(h)) (raw data of Figures 4 and 3 was listed in supplementary files).

### 3.4. Oxidative Stress and Mitochondrial Dynamics Disorders to CIRP in HK-2 Cells

**Figure 5:**
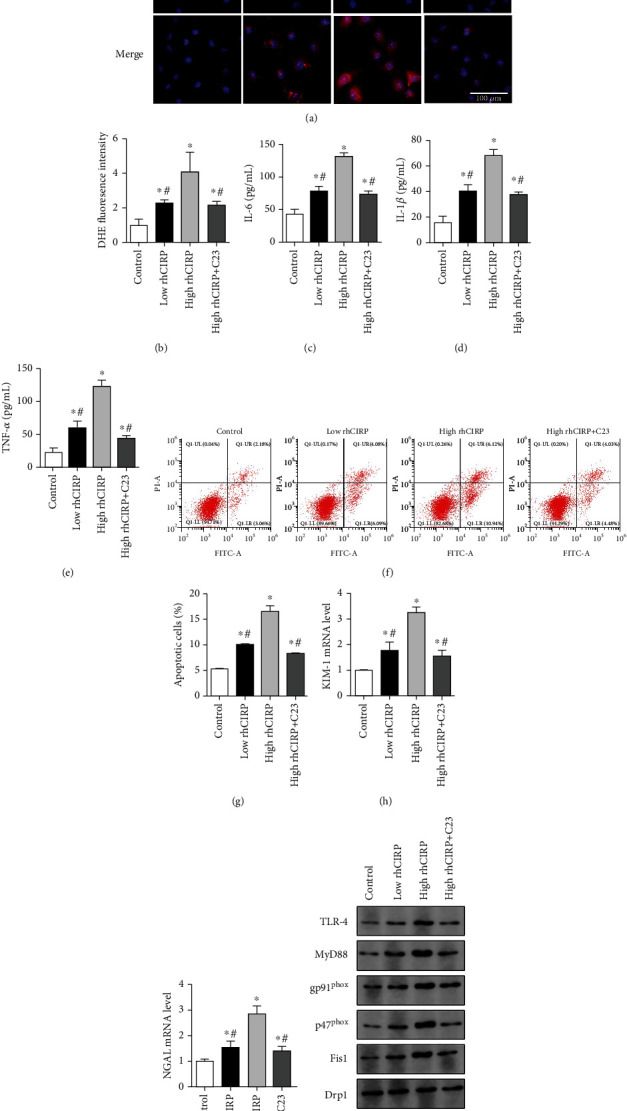
CIRP mediated oxidative stress and mitochondrial dynamics disorder in HK-2 cells. (a) Renal DHE staining (red) and counterstaining DAPI (blue), magnification 400x. (b) Renal DHE fluorescence intensity in each group. (c–e) Expression of inflammatory factors IL-6, IL-1*β*, and TNF-*α* in the medium. (f, g) Flow cytometry analysis of HK-2 cell apoptosis. (h, i) The mRNA levels of KIM-1 and NGAL. (j) Western blot analysis of NADPH oxidase and mitochondrial proteins, rhCIRP, and recombinant human CIRP protein. DHE: dihydroethidium; NADPH: nicotinamide adenine dinucleotide phosphate. ^∗^*P* < 0.05 vs. control; ^#^*P* < 0.05 vs. high rhCIRP. (a) Scale bars: 100 *μ*m.

Different concentrations of rhCIRP or C23 were used to treat HK-2 cells and verify the effect of CIRP. Intracellular ROS levels increased with the concentration of rhCIRP. DHE fluorescence intensity in the high rhCIRP group was 4.1 times that of the control group and 1.8 times that of the low rhCIRP group (*P* < 0.05, Figures 5(a) and 5(b)). Furthermore, rhCIRP activated the TLR-4/MyD88 signaling pathway and upregulated the expression of gp91^phox^ and P47^phox^ (Figure 5(j)). The mitochondrial dynamics disorder was induced by rhCIRP through upregulation of Fis1 and Drp1 and downregulation of Mfn2. Additionally, rhCIRP significantly promoted the secretion of inflammatory factors (Figures 5(c)–5(e)) and aggravated the apoptosis of HK-2 cells (Figures 5(f) and 5(g)). Compared to the control group, the mRNA expression levels of the renal injury markers, KIM-1 and NAGL, increased significantly after stimulation by rhCIRP (Figures 5(h) and 5(i)) (raw data of Figure 5 was listed in supplementary files). However, C23 alleviated CIRP-mediated oxidative stress and mitochondrial dysfunction, inhibited the secretion of inflammatory factors, and reduced apoptosis.

## 4. Discussion

**Figure 6:**
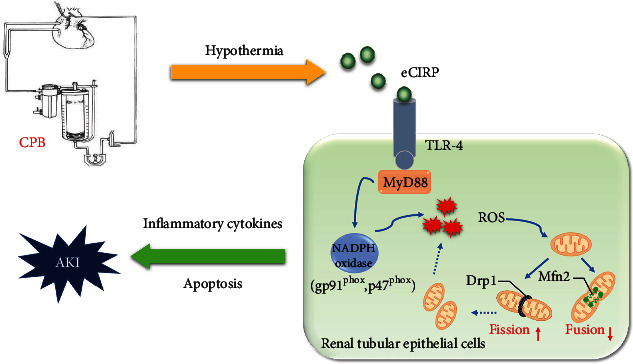
Putative mechanism of CIRP in cardiac surgery-associated acute kidney injury. During cardiac surgery, CIRP is secreted into the circulation in response to hypothermia and hemodynamics change. Extracellular CIRP promotes the expression of NADPH oxidase in renal tubular epithelial cells via the TLR-4/MyD88 pathway and aggravates intracellular oxidative stress. ROS accumulation induces mitochondrial dynamics disorder, which ultimately increases apoptosis and promotes AKI. CIR: cold-inducible RNA-binding protein; NADPH: nicotinamide adenine dinucleotide phosphate; ROS: reactive oxygen species.

CSA-AKI is a common and serious complication associated with cardiac surgery that increases mortality and prolongs hospitalization. In the present study, serum CIRP expression was significantly increased after CPB and was positively correlated with CPB time duration. Patients with high CIRP expression had a high risk of postoperative AKI. Animal models and cell experiments further confirmed that secreted CIRP could promote expression of NADPH oxidase through the TLR-4/MyD88 signaling pathway, aggravate intracellular oxidative stress, mediate a mitochondrial dynamics disorder, and ultimately increase apoptosis (Figure 6).

In mammalian cells, the transcription levels of CIRP peak at mild-to-moderate hypothermia (about 28-32°C) [21, 22]. During cardiac surgeries, a large amount of CIRP is secreted into the circulation due to changes in hemodynamics and body temperature. As a novel DAMP, eCIRP has attracted increasing attention in the field of aseptic inflammation caused by CPB. Chen et al. included 31 patients who underwent cardiac surgeries and found that CIRP expression significantly increased at 6 hours after CPB and gradually returned to normal levels after 5 days post-CPB [23]. Liu et al. established a rat deep hypothermic circulatory arrest model and showed that CIRP was enhanced in microglia aggravated neuronal injury via the Brd2-NF-*κ*B signaling pathway [24]. However, the function of CIRP in CSA-AKI remains unknown. In this study, CIRP expression in patients with CPB was 4.3 times higher than that in patients without CPB, and the risk of AKI in stages 2-3 increased significantly in patients with high CIRP expression. It should be noted that the patients with stage 1 AKI usually recovered before patient discharge, while the patients with stage 2-3 AKI were more likely to require continuous support with renal replacement therapy or develop end-stage kidney disease [2, 25]. Moreover, rates of short-term and long-term mortality associated with AKI increased proportionally with higher disease severity stages [26]. Thus, CSA-AKI prediction may use serum CIRP as a potential biomarker.

Studies have shown that eCIRP not only affects immune cells such as macrophages [10], lymphocytes [27], and neutrophils [28] but also elevates ROS levels and promotes inflammatory response in endothelial [29] and epithelial [30] cells. Recombinant CIRP can directly induce NADPH oxidase activation in mouse lung vascular endothelial cells and increase ROS production to aggravate lung injury [29]. NADPH oxidase consists of two transmembrane proteins including gp91^phox^ and p22^phox^ and four cytosolic proteins including P47^phox^, p67^phox^, p40^phox^, and Rac2, of which gp91^phox^ is the catalytic subunit of NADPH oxidase [31]. In our study, the renal tissues in the CPB group had a higher expression of gp91^phox^ and P47^phox^ than the sham group, which was consistent with the oxidative stress levels. The infiltrations of inflammatory cells in the renal cortex and outer medulla in the CPB group were both higher than the sham group. Furthermore, rhCIRP upregulated the expressions of gp91^phox^ and P47^phox^ in the HK-2 cells, elevated ROS levels, promoted inflammatory factor secretion, and ultimately induced apoptosis. However, inhibition of CIRP expression effectively downregulated the oxidative stress in both tissues and cells.

Increasing evidence has indicated that cellular redox homeostasis is related to mitochondrial dynamics [32]. Under normal conditions, mitochondrial dynamics are coordinated by fission and fusion proteins with regard to amount, activity, and localization. High ROS levels can induce overexpression of fission-related protein Drp1 and mediate mitochondrial fragmentation in a drosophila wound-healing model [33]. Similarly, increased myocardial lipid uptake could elevate mitochondrial ROS generation to induce Drp1 posttranslational modification and mitochondrial fission [34]. Conversely, fragmented mitochondrial morphology and greater ROS levels were evident in the cells devoid of Mfn2 [35]. Exogenous H_2_O_2_ stimulation could induce the ubiquitination of Mfn2 and increase mitochondrial fragmentation in fibroblasts [36]. The mitochondria become the major target and site of ROS damage and production, respectively. Disorders of mitochondrial dynamics and intracellular ROS may accelerate each other. ROS accumulation can trigger mitochondrial fragmentation, swelling, or shortening [37, 38], whereas excessive mitochondrial fragmentation can also in turn increase mitochondrial ROS production. Eventually, high levels of ROS can induce the secretion of inflammatory mediators, such as NLRP3 inflammasome [39], NF-*κ*B [40], and peroxiredoxin-2 [41], among others. In the present study, CIRP promoted the levels of ROS both in renal tissues and cells and further increased Drp1 and Fis1 expression, while reducing Mfn2 expression. Blockage of CIRP expression could effectively reduce the levels of ROS and attenuate mitochondrial dynamics disorders. It is worth noting that the concentration of CIRP serum did not change much at 0 and 4 hours after CPB, but the expression levels of inflammatory factors continued to rise. Therefore, our findings suggest that eCIRP may trigger an imbalance of redox homeostasis and mitochondrial dynamics, resulting in a cascade of inflammatory responses. Further research is required to confirm this finding.

It should be noted that there were some differences between the rat model and clinical model in terms of priming volume, pressure, and temperature during CPB. Since the priming volume cannot be further reduced, excessive volume might increase the risk of postoperative AKI. This may be a shortcoming of the rat CPB model in this study.

## 5. Conclusions

AKI associated with oxidative stress and inflammation induced by CPB is a common and serious cardiac surgery complication. This study demonstrated that the expression of serum CIRP was significantly increased during CPB and was positively correlated with the CPB time duration. Elevated CIRP could further mediate oxidative stress and mitochondrial dynamics disorders in the kidney and promote postoperative AKI. Intervention targeting of CIRP is a potential strategy for the prevention of CSA-AKI.

## Data Availability

The data that support the findings of the present study are available from the corresponding author upon reasonable request.

## Abbreviations

AKI: Acute kidney injury
BUN: Blood urea nitrogen
CABG: Coronary artery bypass grafting
CIRP: Cold-inducible RNA-binding protein
CPB: Cardiopulmonary bypass
CSA-AKI: Cardiac surgery-associated acute kidney injury
*ΔΔ*Ct: Comparative-Ct method
DAMP: Damage-associated molecular pattern
DHE: Dihydroethidium
DMEM: Dulbecco's modified Eagle's medium
Drp1: Dynamin-related protein 1
eCIRP: Extracellular cold-inducible RNA-binding protein
ELISA: Enzyme-linked immunosorbent assays
FBS: Fetal bovine serum
Fis1: Fission 1
G: Gauge
GSH-Px: Glutathione peroxidase
H&E: Hematoxylin and eosin
HK-2: Human Kidney 2
HRP: Horseradish peroxidase
ICU: Intensive care unit
IL-1*β*: Interleukin 1 beta
IL-6: Interleukin 6
IRI: Perioperative renal ischemia-reperfusion injury
KDIGO: Kidney Disease Improving Global Outcomes Definition and Staging
KIM-1: Kidney injury molecule 1
MDA: Malonaldehyde
NADPH: Nicotinamide adenine dinucleotide phosphate
NGAL: Neutrophil gelatinase-associated lipocalin
Pen/strep: Penicillin/streptomycin
PVDF: Polyvinylidene fluoride
rhCIRP: Recombinant human CIRP protein
ROS: Intracellular reactive oxygen species
RT-PCR: Reverse transcription polymerase chain reaction
Scr: Serum creatinine
SDS-PAGE: Sodium dodecylsulfate-polyacrylamide gel electrophoresis
SOD: Superoxide dismutase
TNF: Tumor necrosis factor alpha.

## Supplementary Materials

Supplementary Materials The supplementary files include baseline characteristics of the study patients and raw data of animal and cellular experiments. Table 1 displays baseline characteristics of the study patients. Table 2 displays raw data of Figures 2(b) and 2(c). Table 3 displays raw data of Figures 2(d) and 2(e). Table 4 displays raw data of Figures 2(f) and 2(g). Table 5 displays raw data of Figures 2(j) and 2(k). Table 6 displays raw data of Figures 4(f)–4(h). Table 7 displays raw data of Figures 3(a) and 3(b). Table 8 displays raw data of Figures 3(c) and 3(d). Table 9 displays raw data of Figures 3(e) and 3(f). Table 10 displays raw data of Figures 5(c)–5(e). Table 11 displays raw data of Figure 5(g). Table 12 displays raw data of Figures 5(h) and 5(i).
