# Supplementary material for: Change in Oxidative Stress and Mitochondrial Dynamics in Response to Elevated Cold-Inducible RNA-Binding Protein in Cardiac Surgery-Associated Acute Kidney Injury
Source: Oxid Med Cell Longev. 2022 Jul 9;2022:3576892. doi: 10.1155/2022/3576892 (PMC9288297; doi:10.1155/2022/3576892)
Supplement: Supplementary Materials — The supplementary files include baseline characteristics of the study patients and raw data of animal and cellular experiments. Table 1 displays baseline characteristics of the study patients. Table 2 displays raw data of Figures 2(b) and 2(c). Table 3 displays raw data of Figures 2(d) and 2(e). Table 4 displays raw data of Figures 2(f) and 2(g). Table 5 displays raw data of Figures 2(j) and 2(k). Table 6 displays raw data of Figures 4(f)–4(h). Table 7 displays raw data of Figures 3(a) and 3(b). Table 8 displays raw data of Figures 3(c) and 3(d). Table 9 displays raw data of Figures 3(e) and 3(f). Table 10 displays raw data of Figures 5(c)–5(e). Table 11 displays raw data of Figure 5(g). Table 12 displays raw data of Figures 5(h) and 5(i). [file 3576892.f1.docx]

**Supplementary Files**

**Table 1. Baseline characteristics of the study patients**

| Variables | All patients,  n=292 | CPB,  n=249 | No CPB,  n=43 | *P*-value |
| --- | --- | --- | --- | --- |
| Age (years) ^*^ | 53(42, 61) | 52(39,60) | 61(52,66) | <0.001 |
| Male (%) | 65 | 64 | 74 | 0.164 |
| BMI (Kg/m^2^) ^*^ | 23(21, 25) | 23(20,25) | 24(23,26) | 0.004 |
| Ever smoked (%) | 46 | 43 | 61 | 0.110 |
| Hypertension (%) | 36 | 34 | 47 | 0.128 |
| Prior myocardial infarction(%) | 16 | 9 | 56 | <0.001 |
| Unstable angina (%) | 20 | 10 | 74 | <0.001 |
| NYHA III/IV (%) | 57 | 57 | 54 | 0.665 |
| LVEF (%)^*^ | 62(53, 68) | 62(53,68) | 65(52,79) | 0.132 |
| Preoperative creatinine (μmol/L) ^*^ | 63(53, 74) | 63(53,74) | 65(52,79) | 0.461 |
| Preoperative eGFR (mL/min/1.73m^2^) ^*^ | 103(94, 117) | 104(94,117) | 101(94,110) | 0.118 |

BMI, body mass index; NYHA, New York Heart Association; LVEF, left ventricular ejection fraction; eGFR, estimated glomerular filtration rate.

*Variables are presented as median (25th, 75th percentiles).

**Table 2. Raw data of Fig 2B and 2C**

| Scr 0h (μmol/L) | | | Scr 4h (μmol/L) | | |
| --- | --- | --- | --- | --- | --- |
| Sham | CPB | CPB+C23 | Sham | CPB | CPB+C23 |
| 57.143 | 253.759 | 48.120 | 48.120 | 122.932 | 82.331 |
| 31.955 | 244.737 | 130.075 | 59.398 | 185.338 | 89.850 |
| 58.647 | 90.451 | 96.992 | 62.782 | 156.391 | 128.947 |
| 65.038 | 67.669 | 80.451 | 74.812 | 156.015 | 81.203 |
| 91.353 | 99.624 | 128.947 | 86.090 | 128.947 | 128.195 |
|  | 100.752 | 46.241 |  | 90.602 | 88.346 |
|  |  | 79.323 |  |  | 52.632 |

**Table 3. Raw data of Fig 2D and 2E**

| BUN 0h (mg/dL) | | | BUN 4h (mg/dL) | | |
| --- | --- | --- | --- | --- | --- |
| Sham | CPB | CPB+C23 | Sham | CPB | CPB+C23 |
| 26.637 | 45.433 | 37.585 | 33.660 | 75.245 | 65.299 |
| 32.231 | 34.991 | 46.930 | 30.956 | 67.283 | 61.472 |
| 28.743 | 35.890 | 49.245 | 19.439 | 65.849 | 60.403 |
| 24.213 | 51.458 | 39.736 | 27.722 | 73.671 | 64.528 |
| 24.683 | 41.925 | 39.309 | 37.920 | 63.509 | 68.838 |
|  | 43.925 | 38.528 |  | 75.170 | 60.302 |
|  |  | 42.375 |  |  | 57.763 |

**Table 4. Raw data of Fig 2F and 2G**

| CIRP 0h (pg/mL) | | | CIRP 4h (pg/mL) | | |
| --- | --- | --- | --- | --- | --- |
| Sham | CPB | CPB+C23 | Sham | CPB | CPB+C23 |
| 58.17 | 138.17 | 85.77 | 18.80 | 135.61 | 35.96 |
| 47.59 | 65.30 | 59.36 | 76.09 | 55.30 | 52.28 |
| 79.71 | 152.28 | 35.96 | 123.83 | 139.45 | 80.92 |
| 105.43 | 109.15 | 41.75 | 55.81 | 148.44 | 82.13 |
| 29.05 | 290.51 | 71.28 | 32.50 | 310.84 | 22.20 |
|  | 195.82 | 60.54 |  | 240.93 | 38.27 |
|  |  | 26.76 |  |  | 45.25 |

**Table 5. Raw data of Fig 2J and 2K**

| Renal KIM-1 mRNA level | | | Renal NGAL mRNA level | | |
| --- | --- | --- | --- | --- | --- |
| Sham | CPB | CPB+C23 | Sham | CPB | CPB+C23 |
| 1.000000 | 10.489400 | 1.638239 | 1.000000 | 8.539219 | 1.482215 |
| 1.914306 | 19.197880 | 2.678869 | 2.306030 | 13.693880 | 2.316928 |
| 1.351480 | 14.539350 | 3.833898 | 1.589899 | 10.103840 | 4.297763 |
| 1.139362 | 3.639722 | 8.298194 | 2.046875 | 2.770499 | 6.220450 |
| 0.5687224 | 4.955293 | 4.355910 | 0.6143989 | 5.637797 | 2.995324 |
|  | 13.163290 | 5.217956 |  | 6.906844 | 4.885903 |
|  |  | 3.207743 |  |  | 3.900166 |

**Table 6. Raw data of Fig 3F-H**

| MDA in the kidney (nmol/mg prot) | | | SOD in the kidney (U/mg prot) | | |
| --- | --- | --- | --- | --- | --- |
| Sham | CPB | CPB+C23 | Sham | CPB | CPB+C23 |
| 0.6788313 | 2.603817 | 0.7019302 | 232.264500 | 63.121430 | 130.429600 |
| 0.8246754 | 2.366943 | 1.044008 | 195.560300 | 89.111630 | 89.422970 |
| 0.5155779 | 1.857103 | 1.145333 | 264.773900 | 63.096280 | 106.559100 |
| 0.4546846 | 2.539366 | 1.409547 | 179.254300 | 80.239110 | 131.511900 |
| 0.9186926 | 2.127757 | 1.113924 | 191.489700 | 90.654040 | 153.591400 |
|  | 2.044747 | 1.602910 |  | 52.609840 | 123.619100 |
|  |  | 1.450140 |  |  | 104.259300 |

| GSH-PX in the kidney (U/mg prot) | | |  | | |
| --- | --- | --- | --- | --- | --- |
| Sham | CPB | CPB+C23 |  |  |  |
| 13.808520 | 5.016897 | 5.370381 |  |  |  |
| 12.674620 | 3.127102 | 4.043487 |  |  |  |
| 15.294540 | 4.226842 | 6.400666 |  |  |  |
| 11.869570 | 6.118167 | 8.028290 |  |  |  |
| 10.405650 | 4.443619 | 10.233100 |  |  |  |
|  | 6.114237 | 8.752584 |  |  |  |
|  |  | 7.277470 |  |  |  |

**Table 7. Raw data of Fig 4A and 4B**

| IL- 6 0h after CPB (pg/mL) | | | IL- 6 4h after CPB (pg/mL) | | |
| --- | --- | --- | --- | --- | --- |
| Sham | CPB | CPB+C23 | Sham | CPB | CPB+C23 |
| 53.48 | 5.10 | 3.01 | 28.14 | 227.91 | 88.92 |
| 198.91 | 207.81 | 3.01 | 218.96 | 495.33 | 93.49 |
| 55.60 | 72.63 | 0.93 | 3.01 | 610.28 | 122.11 |
| 114.09 | 261.67 | 3.01 | 259.41 | 404.78 | 241.37 |
| 3.01 | 102.65 | 0.93 | 3.01 | 68.36 | 122.11 |
|  | 28.14 | 3.01 |  | 300.34 | 268.46 |
|  |  | 0.93 |  |  | 195.38 |

**Table 8. Raw data of Fig 4C and 4D**

| IL-1β 0h after CPB (pg/mL) | | | IL-1β 4h after CPB (pg/mL) | | |
| --- | --- | --- | --- | --- | --- |
| Sham | CPB | CPB+C23 | Sham | CPB | CPB+C23 |
| 11.44 | 28.30 | 3.83 | 32.93 | 525.02 | 448.79 |
| 28.30 | 84.64 | 3.83 | 160.87 | 2072.09 | 247.43 |
| 5.35 | 17.55 | 2.32 | 99.00 | 1893.98 | 267.82 |
| 56.25 | 67.24 | 3.83 | 116.71 | 469.53 | 739.76 |
| 25.22 | 68.81 | 5.35 | 26.76 | 570.39 | 336.76 |
|  | 8.40 | 2.32 |  | 1408.33 | 391.22 |
|  |  | 0.80 |  |  | 197.13 |

**Table 9. Raw data of Fig 4E and 4F**

| TNF-α 0h after CPB (pg/mL) | | | TNF-α 4h after CPB (pg/mL) | | |
| --- | --- | --- | --- | --- | --- |
| Sham | CPB | CPB+C23 | Sham | CPB | CPB+C23 |
| 2.57 | 47.92 | 12.85 | 88.01 | 186.97 | 290.43 |
| 6.21 | 46.56 | 19.35 | 24.33 | 193.74 | 163.22 |
| 11.94 | 131.03 | 31.10 | 11.49 | 479.75 | 281.12 |
| 9.10 | 20.89 | 129.99 | 20.63 | 540.73 | 223.56 |
| 3.12 | 58.22 | 20.37 | 7.01 | 314.14 | 170.09 |
|  | 20.63 | 23.26 |  | 501.96 | 108.59 |
|  |  | 46.90 |  |  | 179.45 |

**Table 10. Raw data of Fig 5C-E**

| IL- 6 (pg/mL) | | | | IL-1β (pg/mL) | | | |
| --- | --- | --- | --- | --- | --- | --- | --- |
| Control | Low rhCIRP | High rhCIRP | High rhCIRP+C23 | Control | Low rhCIRP | High rhCIRP | High rhCIRP+C23 |
| 44.576230 | 73.285140 | 136.962400 | 68.289580 | 10.430840 | 35.456380 | 63.283600 | 40.328270 |
| 34.808080 | 85.015430 | 130.827900 | 77.953630 | 20.037250 | 40.163890 | 72.552550 | 36.135720 |
| 48.751430 | 79.839780 | 126.389100 | 75.277830 | 16.308040 | 45.861440 | 70.066760 | 36.593730 |

| TNF-α (pg/mL) | | | |  | | | |
| --- | --- | --- | --- | --- | --- | --- | --- |
| Control | Low rhCIRP | High rhCIRP | High rhCIRP+C23 |  |  |  |  |
| 14.575580 | 53.632400 | 130.847600 | 44.722060 |  |  |  |  |
| 28.455490 | 58.255990 | 110.608000 | 39.186420 |  |  |  |  |
| 21.986960 | 69.616490 | 126.912900 | 44.546750 |  |  |  |  |

**Table 11. Raw data of Fig 5G**

| Apoptotic cells (%) | | | |  | | | |
| --- | --- | --- | --- | --- | --- | --- | --- |
| Control | Low rhCIRP | High rhCIRP | High rhCIRP+C23 |  |  |  |  |
| 5.38 | 9.90 | 17.06 | 8.27 |  |  |  |  |
| 5.24 | 10.17 | 17.61 | 8.66 |  |  |  |  |
| 5.17 | 10.43 | 15.51 | 8.51 |  |  |  |  |

**Table 12. Raw data of Fig 5H and 5I**

| KIM-1 mRNA level | | | | NGAL mRNA level | | | |
| --- | --- | --- | --- | --- | --- | --- | --- |
| Control | Low rhCIRP | High rhCIRP | High rhCIRP+C23 | Control | Low rhCIRP | High rhCIRP | High rhCIRP+C23 |
| 1.000000 | 1.467477 | 3.246071 | 1.762635 | 1.000000 | 1.517985 | 3.108308 | 1.568111 |
| 1.028568 | 1.724960 | 3.013218 | 1.329614 | 1.099889 | 1.788142 | 2.503158 | 1.236832 |
| 0.9626595 | 2.079247 | 3.422457 | 1.542971 | 0.952629 | 1.312913 | 2.766132 | 1.367698 |
